# Filamentous fungal biofilms: Conserved and unique aspects of extracellular matrix composition, mechanisms of drug resistance and regulatory networks in *Aspergillus fumigatus*

**DOI:** 10.1038/s41522-022-00347-3

**Published:** 2022-10-19

**Authors:** Shuai Liu, Francois Le Mauff, Donald C. Sheppard, Shizhu Zhang

**Affiliations:** 1 Jiangsu Key Laboratory for Microbes and Functional Genomics, Jiangsu Engineering and Technology Research Center for Microbiology, College of Life Sciences, Nanjing Normal University, Nanjing, China; 2 Department of Microbiology and Immunology, Faculty of Medicine, McGill University, Montreal, QC Canada; 3 Infectious Disease and Immunity in Global Health, Research Institute of McGill University Health Center, Montreal, QC Canada; 4 McGill Interdisciplinary Initiative in Infection and Immunity, Montreal, QC Canada

**Keywords:** Biofilms, Pathogens

## Abstract

The filamentous fungus *Aspergillus fumigatus* is an ubiquitous mold that can cause invasive pulmonary infections in immunocompromised patients. Within the lung, *A. fumigatus* forms biofilms that can enhance resistance to antifungals and immune defenses, highlighting the importance of defining the mechanisms underlying biofilm development and associated emergent properties. *A*. *fumigatus* biofilms display a morphology and architecture that is distinct from bacterial and yeast biofilms. Moreover, *A. fumigatus* biofilms display unique characteristics in the composition of their extracellular matrix (ECM) and the regulatory networks governing biofilm formation. This review will discuss our current understanding of the form and function of *A. fumigatus* biofilms, including the unique components of ECM matrix, potential drug resistance mechanisms, the regulatory networks governing *A. fumigatus* biofilm formation, and potential therapeutics targeting these structures.

## Introduction

Biofilms are structured microbial communities surrounded by an extracellular matrix (ECM). Distinct from their free-living counterparts, the formation of biofilms increased resistance to anti-microbial drugs and the host immune system, which making them very difficult to combat^1–3^. Biofilms have been best studied in bacteria, and more recently in *Candida* species, however, emerging evidence suggests that filamentous fungi also make biofilms. *Aspergillus fumigatus* is the most common airborne filamentous human fungal pathogen, and causes a spectrum of different symptoms including invasive aspergillosis (IA) in immunocompromised hosts and chronic pulmonary aspergillosis and in patients with chronic lung disease^4^. *A. fumigatus* forms biofilms in both acute and chronic infections although the structure and composition of biofilms can vary between these two sets of conditions^5^. *A. fumigatus* can also colonize the airways of patients with cystic fibrosis (CF) patients, but biofilm formation in this condition has not been studied^6,7^.

Like biofilms in bacteria and yeast, the biofilms of *A. fumigatus* provide protection from antifungal therapy and host immune defenses^8,9^. Recent clinical trials highlight that the mortality of invasive aspergillosis remains as high as 30%, although treatment with the current antifungal agents^10,11^. Biofilm-mediated antifungal resistance is likely a contributing factor to the antifungal treatment failures observed in vivo with *A. fumigatus* isolates that are susceptible to antifungal agents by in vitro antifungal susceptibility testing^12–14^.

The composition of *A. fumigatus* biofilms is distinct from yeast biofilms. The *Candida* biofilms consisted of a dense network of mixture morphological forms, including yeast cells, hyphae and pseudohyphae^15^. In comparison, interconnected, branched multinucleate vegetative hyphae are the main type of cells within *A. fumigatus* biofilms^8^. Three-dimensional surface plot analysis has revealed that spatially ordered hyphae, well-structured hyphal channels and vertical hyphal growth are characteristics of the *A. fumigatus* biofilms^16,17^. Recent studies revealed that these specific features of filamentous fungal biofilms morphologies might play a role in fungal drug resistance and the virulence of *A. fumigatus*^17,18^. In addition, the components of ECM, the mechanisms of drug resistance and the regulatory network governing *A. fumigatus* biofilms are also likely different than in bacteria and yeast. This review will summarize the current state of our knowledge on *A. fumigatus* biofilms architecture, formation, function, and the potential for the development of therapeutics targeting *A. fumigatus* biofilms.

### GAG, a unique glycan within the extracellular matrix in *A*. *fumigatus* biofilms

One of the hallmarks of all biofilms is the presence of extracellular matrix. This extracellular matrix has diverse functions, including mediating surface adherence, as well as enhancing resistance to antifungal agents and host defenses^9,19,20^. The matrix of *A. fumigatus* biofilms is mainly composed of extracellular DNA, polyols, proteins, lipids, and exopolysaccharides including α-glucans, galactomannan, and galactosaminogalactan (GAG)^21,22^. Among them, GAG is a critical structural and functional component of the ECM produced both in vitro and in vivo^5,23^. GAG-mediated adherence is crucial for *A. fumigatus* biofilm formation, strains deficient in GAG production are unable to produce extracellular matrix and fail to form adherent biofilms^24–27^.

Heteropolysaccharide GAG is composed of α-1,4-linked of galactose and partially deacetylated N-acetyl galactosamine (GalNAc)^28,29^. The biosynthesis of GAG is mediated by a cluster of five genes on chromosome 3. The *uge3* gene encodes a glucose 4-epimerase mediating production of the nucleotide monosaccharides uridine diphosphate (UDP)-galactopyranose and UDP-GalNAc^24^. Next, putative transmembrane glycosyltransferase encoded by *gtb3*, polymerizes and exports these substrate sugars^26^. Loss of either *uge3* or *gtb3* is associated with a complete loss of GAG synthesis^24,26^. Two glycoside hydrolases, encoded by *ega3* and *sph3*, exhibit specificity for different regions within the GAG polymer^25,27^. The phenotype of an Ega3-deficient mutant has not been reported, however, Sph3 is required for GAG synthesis^25^. The *agd3* gene encodes a secreted polysaccharide deacetylase mediating deacetylation of GalNAc residues within GAG, rendering the polysaccharide polycationic^30,31^. Agd3-deficient mutants of *A. fumigatus* produce fully acetylated, non-adherent GAG that cannot support biofilm formation^30^. The GAG biosynthetic gene cluster exists on the genomes of some plant and human fungal pathogens, but is absent in *Saccharomyces cerevisiae* and fungal pathogens *C. albicans*^30^.

**Fig. 1:**
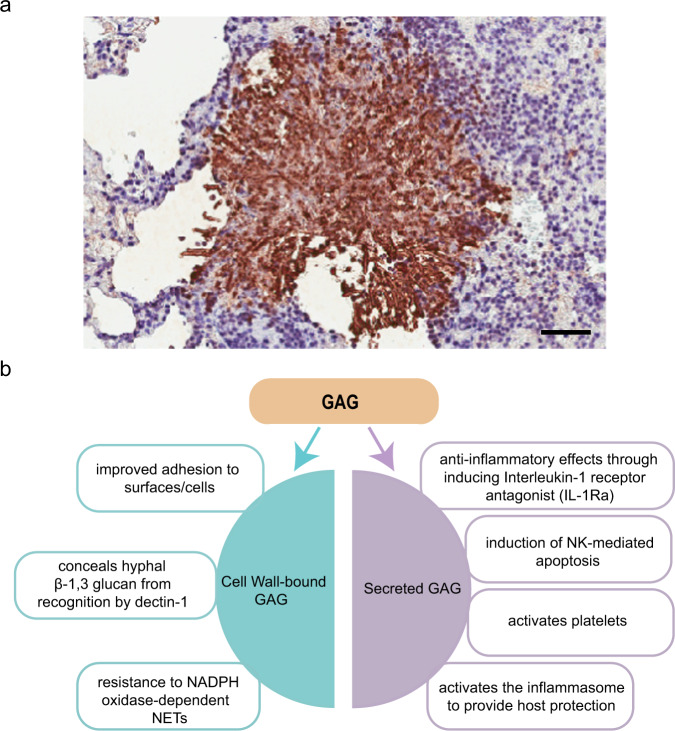
*A. fumigatus* produce GAG in vivo and the multiple roles of GAG in pathogenesis. **a** Immunohistochemistry of pulmonary tissue from an immunocompromised mouse infected with *A. fumigatus* and stained with an anti-galactosaminogalactan antibody. Brown indicates accumulation of galactosaminogalactan-containing biofilm matrix surrounding hyphae growing within pulmonary tissues. **b** Both cell wall-bound GAG and secreted GAG play multiple roles in *Aspergillus* pathogenesis. Scale bar: 150 μm.

As GAG is both covered on the surface of *A. fumigatus* hyphae and secreted as component of ECM, it is therefore at the frontline of the interaction between *A. fumigatus* and the host immune system (Fig. 1a, b). *A. fumigatus* cell wall β-1,3 glucans are recognized as fungal pathogen-associated molecular patterns (PAMPs) by the C-type lectin dectin-1^32^. The cell wall-bound GAG conceals hyphal β-1,3 glucan from recognition by dectin-1. A GAG-deficient ∆*uge3* mutant is associated with increased β-1,3 glucan exposure, enhanced binding of dectin-1 to cell wall β-1,3 glucans, and induced hyperinflammatory response^24,33^. Cell wall-bound GAG also enhances resistance to NADPH oxidase-dependent neutrophil extracellular traps (NETs) which contributes to virulence^34^. In addition, secreted GAG has anti-inflammatory effects through inducing interleukin-1 receptor antagonist (IL-1Ra), which blocks IL-1 signaling^35^, and has been associated with neutrophil apoptosis both in vitro and in vivo^35,36^. GAG is also a direct activator of platelets, which play a key role in the innate immune response^37,38^. More recently, it was reported that GAG activates the NLRP3 inflammasome by binding to ribosome proteins through charge-charge interactions and inhibiting cellular translation mechanisms^26^. Given the multiple effects of GAG in modulating immune responses, GAG is an important fungal virulence factor. GAG-deficient strains showed attenuated virulence in mouse and invertebrate models of invasive aspergillosis^24,30^. A correlation between the ability to produce cell wall GAG and pathogenicity of different *Aspergillus* species has also been observed, underlining the important role of GAG in virulence^34^.

Besides GAG, the roles of other polysaccharides within the ECM of *A. fumigatus* biofilms has not been well determined. Galactomannan, consisting of a mannan core decorated with β-1,5-linked galactofuranose, is dispensable for biofilm formation, although deletion of genes within the galactomannan biosynthetic pathway has been linked to alterations in expression of GAG^39,40^. The *A. fumigatus* biofilm matrix also contains abundant α-glucans, which are also one of the main cell wall polysaccharides of *A. fumigatus*^41,42^. It has been reported that α-1,3-glucans contributes to the aggregation of germinating conidia of *A. fumigatus*^43^. In addition, an α-1,3-glucan deficient mutant *agsB* in *Aspergillus nidulans* formed dispersed mycelial cells under liquid conditions indicated that α-glucans played crucial role in the agglutination of hyphae^44^. However, the role of α-glucans in biofilm formation has not been well defined. Further studies are required to better understand the roles of individual components of the ECM in *A. fumigatus* biofilm development and drug resistance.

### A transcriptional network controls *A. fumigatus* biofilm formation

Studies in *C. albicans* have revealed that at least 54 transcriptional regulators are involved in biofilm formation in this organism^45,46^. Considering the differences in biofilm structure, adhesion factors (GPI proteins *vs* polysaccharides) and cell types in biofilm (mixture of yeast cells, hyphae and pseudohyphae vs hyphae)^47–49^, it has been hypothesized that the regulators of biofilm formation between *C. albicans* and *A. fumigatus* might not well conserved. Using the Reciprocal Best Hits (RBH)^50^, we identified three orthologs of the 54 *C. albicans* biofilms regulators (Flo8, Ada2, and Efg1) have a role in governing *A. fumigatus* biofilms formation. The 28 out of 54 *C. albicans* biofilms regulators have orthologs in *A. fumigatus* but with no reported roles on *A. fumigatus* adhesion and biofilm formation. No orthologs in the genome of *A. fumigatus* were detected for the 23 remaining *C. albicans* biofilms regulators (Supplementary Table 1).

**Fig. 2:**
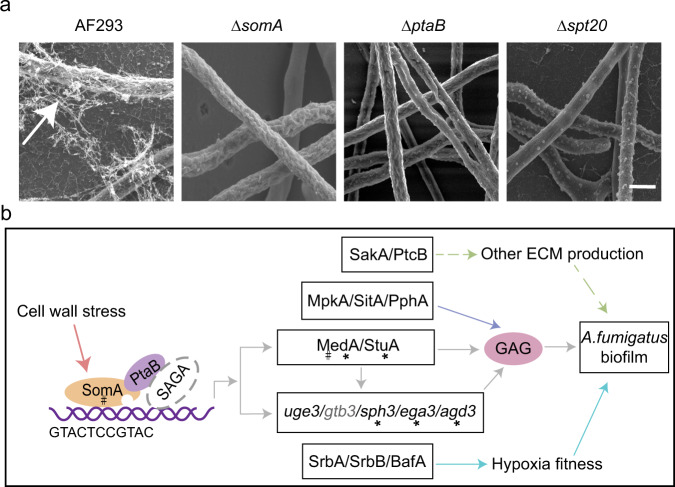
Morphology and regulatory network of *A. fumigatus* biofilms. **a** Scanning electron micrographs of the *A. fumigatus* biofilms. Extensive matrix material, indicated by the arrow, is present on biofilms of *A. fumigatus* wild-type AF293, but is absent in the deletion mutants of *somA, ptaB* and *spt20*. Scale bars: 2 μm. **b** Regulatory network model of *A. fumigatus* biofilms. The asterisks indicated direct binding by SomA *via* to a conserved “GTACTCCGTAC” motif. The # indicated regulators were also required in the GAG production under cell wall stresses condition.

In *A. fumigatus*, several proteins have been identified that play a role in the regulation of adhesion, ECM production, and biofilm formation (Fig. 2a, b). The developmental regulators StuA (the ortholog of Efg1) and MedA positively regulate gene expression in GAG biosynthesis cluster^24,51,52^. The Lim-binding protein PtaB forms a complex with sequence-specific transcription factor SomA (the ortholog of Flo8) which can directly bind to conserved motifs in the *medA* and *stuA* as well as the GAG biosynthesis-related genes *agd3* and *sph3* promoter regions to activate transcription^53^. Recently, some subunits of the transcriptional co-activator Spt-Ada-Gcn5-acetyltransferase (SAGA) complex, including Spt20, Gcn5, AdaB (the ortholog of Ada2), Spt3 and Spt8 were identified as regulators of GAG biosynthesis and biofilm formation^54,55^. Among them, Spt20, a structural subunit of the SAGA complex was found to immunoprecipitate with PtaB, suggesting cooperation between SAGA complex and ptaB/SomA in activating GAG biosynthesis and biofilm formation^55^. Interestingly, the orthologs of *C. albicans* biofilms regulators Flo8, Ada2, and Efg1 are all GAG regulators in *A. fumigatus*. Considering the lack of GAG in *C. albicans*, this observation suggests that fungi can utilize conserved regulators of adhesion and biofilm formation, although the downstream effectors of these pathways are markedly different. In addition to these transcriptional factors, mitogen-activated protein kinases (MAPK) MpkA and SakA as well as phosphatases SitA, PtcB and PphA have been reported to play a role in regulating cell wall compositions, ECM production and biofilm formation in *A. fumigatus*^56–58^. These findings suggest that post-transcriptional pathways are involved in the regulation of *A*. *fumigatus* biofilms.

As with bacteria, the formation of *A. fumigatus* biofilm can be influenced by environmental factors. In bacteria, sub-lethal concentrations of antibiotics commonly induce biofilm formation^59–61^. Antifungal drug caspofungin (inhibiting 1,3-β-glucan synthase) can induce GAG-dependent biofilm formation in *A. fumigatus*^53^. This process is dependent on SomA and MedA, while PtaB and StuA play only a minor role^53^. Recently, it has been reported that light signal regulates the formation of *Aspergillus niger* biofilm by affecting the biosynthesis of melanin and extracellular polysaccharide, although it is not known if this is also the case in *A. fumigatus*^62^, Oxygen tension also significantly affects *A. fumigatus* biofilm development, structure, and function^18^. Hypoxic microenvironments arise during *A. fumigatus* biofilm development. As such, several proteins required for hypoxic fitness can play a role in *A. fumigatus* biofilm development and maturation. Biofilm architecture factor A (BafA), encoded by a small open reading frame within a subtelomeric gene cluster, was found to modify *A. fumigatus* biofilm architecture by increasing the hypoxic fitness^17,63^. Hypoxia-responsive transcription factors SrbA and SrbB, required for *A. fumigatus* growth in low oxygen, can also influence biofilm formation^64^. The loss of SrbA results in an inability to develop a mature biofilm, while the loss of SrbB caused a reduction in overall biofilm biomass and abnormal biofilm structure, albeit to a lesser extent than SrbA disruption^18^. SrbA also plays a role in hyphal polarity and microtubule dynamics, which may be also required for biofilm structure and maturation^65^. Extrapolating from studies in other fungi such as *C. albicans*, it is highly likely that many other regulatory factors involved in *A. fumigatus* biofilm formation remain undiscovered. The availability of a genome-wide collection of *A. fumigatus* transcription-factor-deficient strains provides an opportunity to expand our understanding of regulatory networks for biofilm formation under a range of environmental conditions^66^.

### *A. fumigatus* biofilms and drug resistance

**Fig. 3:**
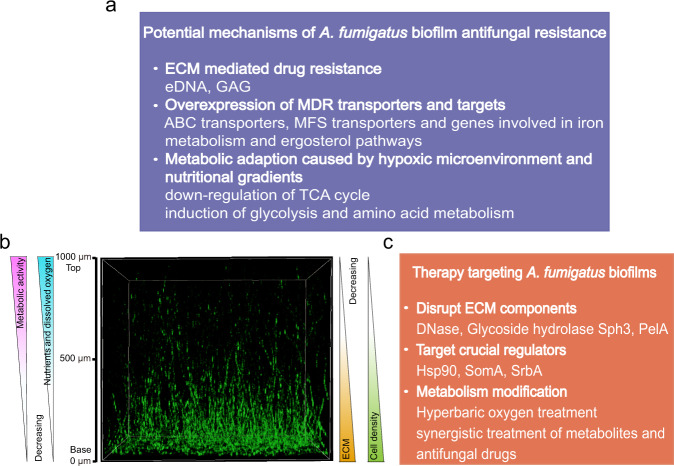
Potential mechanisms of *A. fumigatus* biofilm antifungal resistance and therapeutic approaches to overcome them. **a** Potential antifungal resistance mechanisms within *A. fumigatus* biofilms. **b** 3D view of 24-hour *A. fumigatus* submerged biofilms. Gradients of oxygen, nutrients, metabolic activity, ECM, and cell density result in environmental heterogeneity within *A. fumigatus* biofilms. **c** Approaches to therapies targeting *A. fumigatus* biofilms.

*A. fumigatus* biofilm exhibit greatly increased resistance to all current antifungal drug classes, including azoles, echinocandins, and polyenes when compared to growth under planktonic conditions^19^. As an example, the concentration of voriconazole required to reduce the metabolic activity by 90% (MIC_90_) exceeds 256 mg/mL in mature *A. fumigatus* biofilms^67^. *A. fumigatus* biofilm-associated antifungal resistance is thought to be a consequence of several interrelated factors, including elevated efflux pump activity, ECM production, and altered metabolic states (Fig. 3a).

Several lines of evidence implicate multidrug resistance (MDR) efflux pumps are one of the major contributors to azole resistance of *A. fumigatus* biofilm. An alanine-β-naphthylamine (Ala-Nap) fluorescence assay demonstrated a significant increase in efflux pump activity during the mature of *A. fumigatus* biofilm. Inhibition of efflux pump activity by using an efflux pump inhibitor increases *A. fumigatus* biofilm sensitivity to voriconazole^68^. The *A*. *fumigatus* genome contains 278 predicted major facilitator superfamily (MFS) transporters and 49 predicted ABC transporters, of which 35 are putative multidrug permeases^69^. Only a few of these transporters have been experimentally linked *A. fumigatus* drug resistance. An increased transcript level of *mdr4*, which encodes an ABC transporter, was observed during the development of *A. fumigatus* biofilm. This increase in *mdr4* transcription was coincidental with a significant increased drug resistance in biofilm mature^68^. Additionally, the expression of ABC transporters *cdr1B* and *mdr1* was significantly higher in some azole-resistant strains. The lack of *cdr1B* and *mdr1* largely reduced drug resistance in both wild type *A. fumigatus* and their respective azole-resistant strains^70,71^. However, whether Mdr1 and Cdr1B contributes to *A*. *fumigatus* biofilm drug resistance remains to be defined. Future experiments assessing the expression patterns and the biofilm drug resistance of those MDR efflux pump mutants individually are crucial to better understanding the contribution of MDR efflux pumps on *A. fumigatus* biofilm drug resistance.

ECM production is a fundamental feature of biofilms, providing protection from antimicrobial agents. ECM-mediated drug resistance is common in both bacterial and yeast biofilms^72,73^. The exopolysaccharide GAG is a key component of *A. fumigatus* biofilm ECM. The addition of the GAG specific hydrolase Sph3 significantly increased the activity of the antifungals caspofungin, posaconazole and amphotericin B against 9 h *A*. *fumigatus* biofilms^74^. While this result suggests a role for GAG in antifungal resistance, these data contrast with the observation that GAG-deficient hyphae of the Δ*uge3* null mutant did not exhibit increased susceptibility to antifungals when grown under biofilm-forming conditions^18^. The mechanisms underlying these seemingly contradictory results remain undefined, however it is possible deletion of *uge3* may result in compensatory upregulation of other matrix or cell wall components that enhance antifungal resistance. Extracellular DNA (eDNA) is an important component of biofilm ECM of both fungal and bacterial biofilms^75–77^. In *C. albicans*, eDNA contributes to maintenance and stability of mature biofilms and enhances biofilm antifungal resistance^78^. eDNA also plays an important functional role in maintaining biofilm structural and architectural integrity in *A. fumigatus*^20^. As with anti-GAG hydrolases, DNase treatment enhanced *A. fumigatus* biofilm susceptibility to caspofungin and amphotericin B^20^. Taken as a whole, these results suggest that ECM-mediated drug resistance occurs within *A. fumigatus* biofilms, however, the role of individual ECM components in *A. fumigatus* biofilm antifungal resistance needs to be better defined.

The complex structure of the mature *A. fumigatus* biofilm, composed of spatially ordered mycelium, results in the production of gradients of oxygen, nutrients, metabolic activity, ECM and cell density (Fig. 3b). These nutrients and oxygen gradients generate physiological heterogeneity within the biofilms, a phenomenon associated with antimicrobial resistance in bacterial biofilms^79,80^. The occurrence of hypoxic microenvironment is a canonical feature of many bacterial and yeast biofilms^81–83^, and have been observed in *A. fumigatus* biofilms despite the abundant space between hyphae in the biofilm^18^. Low-oxygen stress can result in increased expression of genes associated with iron and sterol metabolism which has been hypothesized to contribute to the azole drug resistance^84–86^. The transcription factor SrbA, which shares common features with the mammalian sterol regulatory element-binding proteins (SREBPs), can coordinate ergosterol biosynthesis and iron metabolism to mediate both the hypoxic response and azole resistance in *A. fumigatus*^64,65^. Interestingly, hypoxic adaptation, sterol metabolism, and azole drug resistance are instead regulated by zinc finger transcriptional factor Upc2 in *C. albicans*^87,88^, which highlights the differences in regulation of drug resistance between *A. fumigatus* and *C. albicans*. In addition, low-oxygen stress can modify primary metabolic pathways, including the down-regulation of the TCA cycle, induction of glycolysis as well as alanine, aspartate, glutamate metabolism^84^. Consistent with this hypothesis, it was recently reported that an alanine aminotransferase, AlaA, was involved in the resistance of *A. fumigatus* biofilms to echinocandin treatment^89^. However, growth of *A. fumigatus* in a low-oxygen environment is not sufficient to promote antifungal drug resistance, which indicated that other features of filamentous fungal biofilms may also be required to contribute to antimicrobial drug resistance^90,91^. Further investigation is required to explore the signals that guide polar hyphal growth in biofilms. Elucidating the role of oxygen gradients, nutrients, and secondary metabolites in biofilm development and how fungi sense those signals are promising areas of future study. Additionally, more work is required to identify and characterize genes and metabolic pathways that confer biofilm antifungal resistance in *A. fumigatus*.

### Therapeutics targeting *Aspergillus* fungal biofilms

The biofilm lifestyle affords fungi with greater resistance to antifungal agents, an improved ability to evade host immune responses and survive in the in vivo environment^92^. Although antifungal drugs treatment is currently the most important and effective measure for the control of fungal infections, biofilm formation can compromise their efficacy^67^. Therefore, there is a critical need to identify antifungals active against fungal biofilms, or develop novel therapeutics that target the process of biofilm formation itself (Fig. 3c).

A number of studies in bacterial biofilms have suggested that disruption of the ECM in combination with antimicrobial therapy can be an effective strategy to combat biofilm-forming organisms^93,94^. Consistent with this strategy, enzymatic degradation of *A. fumigatus* biofilms ECM components eDNA and GAG have been successfully employed to disrupt biofilms, reduce fungal growth and increase antifungal efficacy in vitro and in vivo. The combination of DNase and antifungal drugs can improve the effect of polyenes and echinoctins against mature *A. fumigatus* biofilms in vitro^20^. These data suggest that DNase therapy may be effective in the management of *A. fumigatus* infections. Importantly, DNase is currently used as an adjunct to antibiotic treatment for cystic fibrosis, supporting the potentials of this agent for the clinical development^95^. Two glycoside hydrolases (GH) Sph3 and Ega3 that can cleave GAG were found by studying the GAG biosynthesis pathway. Treatment with Sph3 and Ega3 soluble recombinant GH domains can hydrolyze GAG and disrupt *A. fumigatus* biofilms in vitro^25,27,74^. GH enzymes can also exhibit cross-kingdom activity. *Pseudomonas aeruginosa* can produce a biofilm exopolysaccharide Pel, which is structurally similar to GAG. The soluble recombinant GH domain of PelA (a protein within the Pel biosynthetic pathway) can cleave GAG and disrupt *A. fumigatus* biofilms in vitro^96^. Intratracheal GH prophylaxis improved survival in neutropenic mice, possibly by increasing pulmonary inflammatory responses^97^. Prophylactic Sph3h combined with posaconazole therapy also enhanced the antifungal activity in a neutropenic mouse model of invasive pulmonary aspergillosis^97^. The activity of these agents against established fungal biofilms in animal models have not yet been reported. Collectively these studies suggest that therapies targeting ECM may hold promise as novel therapeutics for invasive aspergillosis.

Modulating the regulators of biofilm formation is another attractive target for the development of anti-biofilm therapies. Molecular chaperone Hsp90 is a key regulator of fungal drug resistance in multiple fungal species^98–101^. Genetic or pharmacologic inhibition of Hsp90 function significantly increased the efficacy of fluconazole in eradicating the biofilm of *C. albicans* in a rat venous catheter infection model^102^. This finding is consistent with the result that Hsp90 positively regulates matrix glucan production, an important carbohydrate for drug resistance of *C. albicans* biofilms^102^. Inhibition of Hsp90 also reduced the resistance of *A. fumigatus* biofilms to echinocandins and azoles in vitro, although, whether Hsp90 regulates *A. fumigatus* biofilms matrix production remains unknown^102^. Other regulators of *A. fumigatus* biofilm development are potential valuable anti-biofilms targets. SomA is a master transcriptional factor required for both GAG production and cell wall stress responses, and lacks an identifiable ortholog in humans, suggesting it might serve as an attractive target for anti-biofilm drug development^53^.

The hypoxic microenvironment within the *A. fumigatus* biofilm is critical for antifungal resistance^18^. Increasing levels of oxygen within biofilms, therefore, has the potential to reduce biofilm-mediated drug resistance. Hyperbaric oxygen treatment (HBOT) has been successfully to enhance the effect of tobramycin against biofilms formed by the bacterial pathogens *Staphylococcus aureus* and *P. aeruginosa*^103,104^. In *A. fumigatus*, HBOT markedly reduced biofilm proliferation in vitro and increased survival time in a chemotherapy murine model of invasive pulmonary aspergillosis, but this treatment failed to synergize with voriconazole or amphotericin B both in vitro and in vivo^105^. In addition to oxygen, other factors such as specific nutrients, secondary metabolites, or host-produced molecules may have the potential to alter metabolic adaptations of biofilm lifestyles, leading to drug resistance. A better understanding of these pathways may open up novel approaches for treating biofilm-associated infections.

## Conclusions

In the last two decades, we have made significant progress on understanding the mechanisms underlying *A*. *fumigatus* biofilm formation and regulation. However, there are many unanswered questions about *Aspergillus* biofilm development and mechanisms of drug resistance. The knowledge derived from bacteria and yeast biofilms cannot all be directly extrapolated to filamentous fungi. An improved understanding of the unique aspects of filamentous fungal biofilm architecture may help open new therapeutic avenues to combat these deadly infections.

## Supplementary information

Supplementary Material

## Data Availability

All data generated or analyzed during this study are included in this published article (and its supplementary information files).
