# Supplementary material for: Filamentous fungal biofilms: Conserved and unique aspects of extracellular matrix composition, mechanisms of drug resistance and regulatory networks in Aspergillus fumigatus
Source: NPJ Biofilms Microbiomes. 2022 Oct 19;8:83. doi: 10.1038/s41522-022-00347-3 (PMC9581972; doi:10.1038/s41522-022-00347-3)
Supplement: Supplementary file 1 — Supplementary Material [file 41522_2022_347_MOESM1_ESM.pdf]

Supplementary Table 1 The *A. fumigatus* orthologs of *C. albicans* biofilms formation transcriptional regulators

Biofilm regulators in *C.albicans*	Orthologs in *A. fumigatus*	Function in *A. fumigatus*	Identities	References
flo8	Afu7g02260 (*somA*)	Regulator of adherence, biofilm formation, development, virulence and cell wall homeostasis	23%	1-3
efg1	Afu2g07900 (*stuA*)	APSES domain transcription factor involved in regulation of conidiophore development, biofilm formation and secondary metabolism	73%	2,4-6
bcr1	Afu3g09820 (*dvrA*)	Regulator of virulence and host cell interactions	71%	7
brg1	Afu3g13870 (*nsdD*)	Regulator of asexual development, mating and putative regulator of cell wall synthesis	68%	8-10
tec1	Afu1g04830 (*abaA*)	Transcriptional activator involved in regulation of conidiation, cell death, autolysis and biogenesis of gliotoxin	48%	11,12
leu3	Afu2g03460 (*leuB*)	The Zn2Cys6-type transcription factor LeuB cross-links regulation of leucine biosynthesis and iron acquisition	43%	13
tup1	Afu6g05150 (*rocA*)	Transcriptional repressor	59%	14
zap1	Afu1g10080 (*zafA*)	The zinc-responsive transcription factor that controls the adaptive response to zinc starvation and virulence	39%	15,16
cas5	Afu1g16590 (*brlA*)	A key activator for developmental initiation and transcription factor of response to nitrogen depletion	48%	6,17,18
rim101	Afu3g11970 (*pacC*)	The pH-responsive PacC transcription factor governs epithelial entry and tissue invasion during pulmonary aspergillosis	67%	19,20

bpr1	Afu1g03210 (*flbD*)	Transcription activator governs conidium formation and development	61%	17
ada2	Afu2g10640 (*adaB*)	A subunit of the SAGA complex, involved in regulation of conidiophore development, biofilm formation and drug resistance	43%	21,22
ace2	Afu3g11250 (*ace2*)	Transcription factor governs pigment production, conidiation and virulence	45%	23
gcn4	Afu4g12470 (*cpcA*)	Regulator of amino acid biosynthesis and virulence	39%	24,25
fcr3	Afu2g02540 (*rmsA*)	Transcription factor governs gliotoxin cluster metabolites	28%	26
gzf3	Afu2g13380 (*areB*)	Regulator involved in nitrogen and carbon metabolism in *Aspergillus nidulans*	89%	27,28
snf5	Afu2g16840	Unknown function	32%	—
try2	Afu3g02380	Unknown function	42%	—
sfp1	Afu1g14750	Unknown function	66%	—
taf14	Afu5g12190	Unknown function	38%	—
war1	Afu7g01640	Unknown function	45%	—
try5	Afu2g16230	Unknown function	73%	—
gal4	Afu3g06740	Unknown function	38%	—
suc1	Afu1g17150	Unknown function	28%	—
rfg1	Afu4g10820	Unknown function	49%	—
zcf28	Afu1g01650	Unknown function	38%	—
zcf32	Afu1g01850	Unknown function	56%	—
zcf34	Afu5g01700	Unknown function	37%	—
ahr1	Afu5g11070	Unknown function	35%	—
dal81	Afu3g08050	Unknown function	32%	—

fgr27	Afu8g04540	Unknown function	29%	—
ndt80	ND	—	—	—
rob1	ND	—	—	—
rfx2	ND	—	—	—
nrg1	ND	—	—	—
try4	ND	—	—	—
try6	ND	—	—	—
als1	ND	—	—	—
als2	ND	—	—	—
als3	ND	—	—	—
hwp1	ND	—	—	—
ugm6	ND	—	—	—
tye7	ND	—	—	—
mss11	ND	—	—	—
czf1	ND	—	—	—
try3	ND	—	—	—
uga33	ND	—	—	—
zcf31	ND	—	—	—
zcf39	ND	—	—	—
zfu2	ND	—	—	—
znc1	ND	—	—	—
meT4	ND	—	—	—
not3	ND	—	—	—
sap6	ND	—	—	—

To detect the orthologs, a method called reciprocal Best Hits (RBH) was used. Orthologs are assumed if two genes each in a different genome find each other as the best hit in the other genome[29].

The descriptions of the orthologs in *A. fumigatus* are based on FungiDB database (http://fungidb.org/fungidb/) release 56, 15 Feb 2022 and NCBI-Pubmed (https://www.ncbi.nlm.nih.gov).

References

1. Lin, C.J., *et al.* Transcription factor SomA is required for adhesion, development and virulence of the human pathogen *Aspergillus fumigatus*. *PLoS Pathog* **11,** e1005205 (2015).
2. Zhang, S., *et al.* PtaB, a lim-domain binding protein in *Aspergillus fumigatus* regulates biofilm formation and conidiation through distinct pathways. *Cell Microbiol* **20，** e12799 (2018).
3. Chen, Y., *et al.* The transcription factor SomA synchronously regulates biofilm formation and cell wall homeostasis in *Aspergillus fumigatus*. *mBio* **11,** e02329-02320 (2020).
4. Gravelat, F.N., *et al. Aspergillus* galactosaminogalactan mediates adherence to host constituents and conceals hyphal beta-glucan from the immune system. *PLoS Pathog* **9,** e1003575 (2013).
5. Sheppard, D.C., *et al.* The *Aspergillus fumigatus* StuA protein governs the up-regulation of a discrete transcriptional program during the acquisition of developmental competence. *Mol Biol Cell* **16,** 5866-5879 (2005).
6. Twumasi-Boateng, K., *et al.* Transcriptional profiling identifies a role for BrlA in the response to nitrogen depletion and for StuA in the regulation of secondary metabolite clusters in *Aspergillus fumigatus*. *Eukaryot Cell* **8,** 104-115 (2009).
7. Ejzykowicz, D.E., *et al.* Role of *Aspergillus fumigatus* DvrA in host cell interactions and virulence. *Eukaryot Cell* **9,** 1432-1440 (2010).
8. Lee, M.-K., *et al.* NsdD is a key repressor of asexual development in *Aspergillus nidulans*. *Genetics* **197,** 159-173 (2014).
9. Szewczyk, E. & Krappmann, S. Conserved regulators of mating are essential for *Aspergillus fumigatus* cleistothecium formation. *Eukaryot Cell* **9**, 774-783 (2010).
10. Lee, M.K., *et al.* Negative regulation and developmental competence in *Aspergillus*. *Sci Rep* **6,** 28874 (2016).
11. Tao, L. & Yu, J.H. AbaA and WetA govern distinct stages of *Aspergillus fumigatus* development. *Microbiology* **157,** 313-326 (2011).
12. Shin, K.S., Kim, Y.H. & Yu, J.H. Proteomic analyses reveal the key roles of BrlA and AbaA in biogenesis of gliotoxin in *Aspergillus fumigatus*. *Biochem Biophys*

Res Commun **463,** 428-433 (2015).

13. Long, N.*, et al.* The Zn2Cys6-type transcription factor LeuB cross-links regulation of leucine biosynthesis and iron acquisition in *Aspergillus fumigatus*. *PLoS Genet* **14,** e1007762 (2018).
14. Johnk, B.*, et al.* SCF ubiquitin ligase F-box protein Fbx15 controls nuclear co-repressor localization, stress response and virulence of the human pathogen *Aspergillus fumigatus*. *PLoS Pathog* **12**, e1005899 (2016).
15. Vicentefranqueira, R.*, et al.* The transcription factor ZafA regulates the homeostatic and adaptive response to zinc starvation in *Aspergillus fumigatus*. *Genes (Basel)* **9**, 318 (2018).
16. Moreno, M.Á.*, et al.* The regulation of zinc homeostasis by the ZafA transcriptional activator is essential for *Aspergillus fumigatus* virulence. *Mol Microbiol* **64,** 1182-1197 (2007).
17. Park, H.S. & Yu, J.H. Developmental regulators in *Aspergillus fumigatus*. *J Microbiol* **54**, 223-231 (2016).
18. Mah, J.H. & Yu, J.H. Upstream and downstream regulation of asexual development in *Aspergillus fumigatus*. *Eukaryot Cell* **5**, 1585-1595 (2006).
19. Bignell, E.*, et al.* The *Aspergillus* pH-responsive transcription factor PacC regulates virulence. *Mol Microbiol* **55**, 1072-1084 (2005).
20. Staff, P.P. Correction: The pH-responsive PacC transcription factor of *Aspergillus fumigatus* governs epithelial entry and tissue invasion during pulmonary aspergillosis. *PLoS Pathog* **11**, e1004802 (2015).
21. Chen, Y.*, et al.* Spt20, a structural subunit of the SAGA complex, regulates *Aspergillus fumigatus* biofilm formation, asexual development, and virulence. *Appl Environ Microbiol* **88**, e0153521 (2022).
22. Furukawa, T.*, et al.* The negative cofactor 2 complex is a key regulator of drug resistance in *Aspergillus fumigatus*. *Nat Commun* **11**, 427 (2020).
23. Ejzykowicz, D.E.*, et al.* The *Aspergillus fumigatus* transcription factor Ace2 governs pigment production, conidiation and virulence. *Mol Microbiol* **72**, 155-169 (2009).
24. Krappmann, S.*, et al.* The *Aspergillus fumigatus* transcriptional activator CpcA contributes significantly to the virulence of this fungal pathogen. *Mol Microbiol* **52,** 785-799 (2004).
25. Lee, I.R., Morrow, C.A. & Fraser, J.A. Nitrogen regulation of virulence in clinically prevalent fungal pathogens. *FEMS Microbiol Lett* **345,** 77-84 (2013).
26. Sekonyela, R.*, et al.* RsmA regulates *Aspergillus fumigatus* gliotoxin cluster metabolites including cyclo(L-Phe-L-Ser), a potential new diagnostic marker for invasive aspergillosis. *PLoS One* **8,** e62591 (2013).
27. Macios, M., Caddick, M.X., Weglenski, P., Scazzocchio, C. & Dzikowska, A. The GATA factors AREA and AREB together with the co-repressor NMRA, negatively regulate arginine catabolism in *Aspergillus nidulans* in response to nitrogen and carbon source. *Fungal Genet Biol* **49,** 189-198 (2012).

28. Chudzicka-Ormaniec, P., *et al.* The role of the GATA transcription factor AreB in regulation of nitrogen and carbon metabolism in *Aspergillus nidulans*. *FEMS Microbiol Lett* **366** (2019).
29. Moreno-Hagelsieb, G. & Latimer, K. Choosing BLAST options for better detection of orthologs as reciprocal best hits. *Bioinformatics* **24**, 319-324 (2008).
